# Mortality and its predictors among people with dementia receiving psychiatric in-patient care

**DOI:** 10.1192/bjo.2025.40

**Published:** 2025-05-09

**Authors:** Oriane E. Marguet, Shanquan Chen, Emad Sidhom, Emma Wolverson, Gregor Russell, George Crowther, Simon R. White, Jonathan Lewis, Rebecca Dunning, Shahrin Hasan, Benjamin R. Underwood

**Affiliations:** Department of Clinical Neurosciences, University of Cambridge, Cambridge, UK; The London School of Hygiene & Tropical Medicine, London, UK; Cambridgeshire and Peterborough NHS Foundation Trust, Cambridge, UK; The Geller Institute of Ageing and Memory, University of West London, London, UK; Humber Teaching NHS Foundation Trust, Hull, UK; Bradford District Care NHS Foundation Trust, Bradford, UK; Leeds and York Partnership NHS Foundation Trust, Leeds, UK; Department of Psychiatry, University of Cambridge, Cambridge, UK; MRC Biostatistics Unit, University of Cambridge, Cambridge, UK; Tees, Esk and Wear Valleys NHS Foundation Trust, Darlington, UK

**Keywords:** Dementia, behavioural and psychological symptoms of dementia, mortality, palliative care

## Abstract

**Background:**

Although dementia is a terminal condition, palliation can be a challenge for clinical services. As dementia progresses, people frequently develop behavioural and psychological symptoms, sometimes so severe they require care in specialist dementia mental health wards. Although these are often a marker of late disease, there has been little research on the mortality of people admitted to these wards.

**Aims:**

We sought to describe the mortality of this group, both on-ward and after discharge, and to investigate clinical features predicting 1-year mortality.

**Method:**

First, we conducted a retrospective analysis of 576 people with dementia admitted to the Cambridgeshire and Peterborough National Health Service (NHS) Foundation Trust dementia wards over an 8-year period. We attempted to identify predictors of mortality and build predictive machine learning models. To investigate deaths occurring during admission, we conducted a second analysis as a retrospective service evaluation involving mental health wards for people with dementia at four NHS trusts, including 1976 admissions over 7 years.

**Results:**

Survival following admission showed high variability, with a median of 1201 days (3.3 years). We were not able to accurately predict those at high risk of death from clinical data. We found that on-ward mortality remains rare but had increased from 3 deaths per year in 2013 to 13 in 2019.

**Conclusions:**

We suggest that arrangements to ensure effective palliation are available on all such wards. It is not clear where discussions around end-of-life care are best placed in the dementia pathway, but we suggest it should be considered at admission.

Prior to commencing this work, we carried out a systematic review of research relating to specialist dementia in-patient units, finding only four papers relating to mortality and only one that looked at mortality following discharge. That is what we found, despite this topic featuring in a Delphi consensus of importance to relatives and staff and evidence that practice on these wards and available resources both vary widely. Previous work has focused on patients with dementia admitted to acute hospitals and, in this setting, models have been described that can predict mortality. No similar research has been published with regard to patients with dementia admitted to psychiatric wards.

## Added value of this study

To our knowledge, our work is the first to describe survival time for people living with dementia following admission to specialised dementia mental health ward settings, and the first to report an increasing number of deaths occurring on these wards over time. We could not use clinical data to predict risk of death with a degree of accuracy for clinical utility.

## Implications of all the available evidence

Our findings suggest that specialised dementia mental health wards should have access to appropriate palliative care support. Although mortality is variable for this patient group, admission provides a point in care where end-of-life discussions should at least be considered.

## Background

Dementia is one of the leading causes of disability and death worldwide and represents a major health and societal challenge.^1^ Ninety per cent of people living with dementia (PLWD) develop neuropsychiatric symptoms, often grouped under the terminology of ‘behavioural and psychological symptoms of dementia’ (BPSD), with these symptoms becoming more frequent in advanced disease.^2,3^ These can take the form of distress, depression, apathy, sleep disorders, aggression, disinhibition or psychosis.^4^ Neuropsychiatric symptoms in PLWD complicate care and are a significant predictor of carer stress and transition to institutional living. When very high levels of distress occur, specialist psychiatric in-patient care in dementia mental health wards (DMHWs) may be required to maintain the safety of PLWD or their carers. PLWD with severe BPSD have a higher mortality compared with patients living with dementia only.^5,6^ However, there is scant literature addressing mortality, either on DMHWs or following discharge, leading to restricted evidence to guide practice regarding end-of-life care for this population.^7^ While clinicians are usually able to identify an imminent death, their accuracy in determining prognosis is lower for more distal events.^8^ Patient survival time tends to be overestimated by clinicians, which may impair discussions around end-of-life care, and when this topic is best placed in the care pathway is not clear.^9^ Although PLWD admitted to dementia mental health units have a terminal diagnosis, previous research has shown that few patients from DMHWs are transferred to hospices (7–9%)^10^ and that there have been few discussions (9%) about resuscitation status with patients and their families.^11^ Failure to recognise the need for end-of-life care can lead to increased discomfort for PLWD due to inadequate treatment of symptoms common at the end of life.^12^ There is evidence suggesting that access to end-of-life care is not straightforward for PLWD in DMHWs.^12,13^ Given the increasing number of PLWD, there is a pressing need for further research in this patient population to ensure that patients have access to appropriate care pathways and die well. In general hospitals, mortality for PLWD has been investigated, with death within 1 year of admission ranging between 30 and 50% at different sites^14,15^; and, in addition, mortality correlated with worse cognitive performance.^16^ One study looking at prognosis for PLWD in acute hospital care developed a multivariate logistic regression model able to predict patients likely to die within 1 year of admission, which has the potential to help stratify patients according to risk of death and therefore provide better access to palliative care when needed.^14^

## Aims

In our study, we examined mortality for patients with dementia requiring psychiatric in-patient care using the anonymised electronic patient database of Cambridgeshire and Peterborough National Health Service (NHS) Foundation Trust (CPFT) over 7 years. We aimed to describe mortality for PLWD admitted to DMHWs, and to investigate whether it is possible to predict the risk of death at the time of admission using machine learning models. If such models can be built, as they have for PLWD admitted to general hospital wards, this would raise the possibility of tailoring care and discussions about palliation for patients based on prognosis. Given the variability in service provision experienced in DMHWs,^7^ we further investigated the number of PLWD dying on these wards and the change in this over time using data from four different DMHWs in the UK, with the aim of better understanding the need for palliative care support for these wards.

## Method

### Study design and participants

To examine longer-term mortality in this population, we conducted a retrospective cohort study using routinely collected data from CPFT. CPFT operates the Clinical Records Anonymisation and Text Extraction (CRATE) system, which extracts data from clinical records and pseudonymises them at patient level.^17^ The database has overarching NHS Research Ethics approval (nos 12/EE/0407, 17/EE/0442 and 22/EE/0264), and this study was further individually approved by the database committee. We used the electronic clinical records from patients admitted between January 2012 and December 2019. Eligible patients were those with a coded diagnosis of dementia using the World Health Organization International Statistical Classification of Diseases (ICD-10)^18^ codes F00-/G30- (Alzheimer’s disease), F01 (vascular dementia), F02 (dementia in other diseases classified elsewhere) and F03 (unspecified dementia), and who had been admitted to CPFT between the dates cited above. There were no specific exclusion criteria. A flow chart of recruitment is available in Supplementary Fig. 1 (available at https://doi.org/10.1192/bjo.2025.40). Our cohort included 576 patients that we followed up to February 2023 to record their death if it happened over that period.

### Data collection

Data were routinely collected by clinicians at the time of patient admission and extracted for the purpose of this study following anonymisation. We examined the following sociodemographic variables: age at admission (in years), sex assigned at birth, marital status, ethnicity and Index of Multiple Deprivation (IMD). We also examined the following clinical features: results at either Addenbrooke’s Cognitive Examination (ACE), mini-ACE or Mini-Mental State Examination (MMSE); Health of the Nation Outcome Scales (HoNOS) total score, as well as scores in individual categories; and diagnosis according to ICD-10 codes. Cognitive tests were conducted at the time of admission to the ward; 28% of patients completed ACE, 33.7% MMSE and 6.3% mini-ACE. We also examined those medications most commonly prescribed to patients in our trust and that are used to treat symptoms of dementia or neuropsychiatric symptoms: memantine, donepezil, rivastigmine, galantamine, risperidone, citalopram, mirtazapine, sertraline and trazodone. These specific medications were selected due to their being considered as both first-line treatments for Alzheimer’s disease and highly prescribed antidepressants. We examined prescriptions given to patients up until their date of admission, to avoid the possibility of reverse causality (that those who lived longer accumulated more prescriptions).

### Kaplan–Meier survival curves

We performed survival time analyses of patients following their date of admission to DMHW and plotted Kaplan–Meier survival curves, with censoring of patients who were still alive at the point of data extraction. We used these to determine the median survival time at which 50% of the sample would be alive, and maximal survival duration.

### Comparing groups of patients

Patients were segregated in two groups based on their mortality: patients who died within 1 year of admission to the ward versus those who survived for longer, because anticipated death within 1 year is a criterion used to identify potential palliative care needs. We included in the second group patients who were still alive. Using the two groups defined above, statistical comparisons were performed on the basis of patients’ sociodemographic characteristics and the clinical variables defined above. Statistical significance was defined as: *P < 0.05, **P < 0.01, ***P < 0.001, ****P < 0.0001. Because samples were non-normally distributed, we used the two-sample Mann–Whitney *U*-test to analyse continuous variables. Groups were described with median and interquartile range using the format (median [IQR]), and boxplots produced in R (version 4.2.3 for Windows; The R Foundation, Vienna, Austria; https://cloud.r-project.org/). For categorical variables, 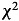 tests of independence were used when sample size was sufficiently large; if the number of data points in each category was too low for the test as indicated by R, a Fisher’s exact test was used. For ordinal data (IMD), median and mode were obtained and Mann–Whitney *U*-tests performed to investigate differences between groups.

### Machine learning

We constructed eight prediction models to investigate whether clinical data available at admission could predict death within 1 year. Our selection of models was designed to explore different approaches to prediction, ranging from simpler linear methods to more complex machine learning algorithms. We included linear discriminant analysis (LDA) and logistic regression (generalised linear model, GLM) as our linear algorithms, chosen for their interpretability and established use in medical prediction tasks. For capture of potential non-linear relationships in the data, we implemented classification and regression trees (CART), *k*-nearest neighbours (KNN), neural networks and naive Bayes classifiers. Additionally, we employed more sophisticated ensemble methods – support vector machines (SVMs) with radial basis function kernels and random forests – known for their ability to model complex interactions between variables and provide typically strong predictive performance in healthcare applications.

To address the challenge of high-dimensional data and potential overfitting, we implemented a feature selection process using recursive feature elimination (RFE) with a random forest algorithm as the estimator. This was performed using the ‘rfe’ function from the ‘caret’ package in R, with ‘method = “rf”’ as the base model. The process began with all available clinical and demographic variables and iteratively removed the least important features based on their contribution to model performance, determined by variable importance scores from the random forest. The initial feature set encompassed demographic variables (age, sex, marital status, ethnicity and IMD), clinical assessments (including cognitive test scores from ACE, mini-ACE or MMSE, along with both total HoNOS and individual category scores), formal diagnosis codes and detailed medication history. The medication data included prescriptions of cognitive enhancers (memantine, donepezil, rivastigmine and galantamine) and psychotropic medications (risperidone, citalopram, mirtazapine, sertraline and trazodone) up to the point of admission.

In preparing the data for model development, we employed the ‘createDataPartition’ function from the ‘caret’ package to perform a stratified random split, allocating 80% of the data (462 patients) to a training data-set and reserving the remaining 20% (114 patients) for final model evaluation. Given the significant class imbalance in our dataset, with only 20.7% of patients dying within 1 year of admission, we applied the Synthetic Minority Over-sampling Technique (SMOTE)^19^ using the ‘SMOTE’ function from the ‘DMwR’ package in R.

The implementation of our models utilised the ‘train’ function from the ‘caret’ package in R, with model-specific configurations. For linear discriminant analysis, we used method = ‘lda’ from the ‘MASS’ package. The logistic regression model employed method = ‘glm’ with family = ‘binomial’. For CART (method = ‘rpart’ from the ‘rpart’ package), we used tuneLength = 10 to optimise the complexity parameter through cross-validation. The KNN implementation (method = ‘knn’ from the ‘class’ package) included tuning of *k*-values from 1 to 20. Our neural network (method = ‘nnet’) architecture used size = 3 hidden units, with decay = 0.1 and maximum iterations set to 1000. The naive Bayes classifier was implemented using method = ‘naive_bayes’ from the ‘naivebayes’ package. The SVM implementation (method = ‘svmRadial’ from the ‘kernlab’ package) used automatic optimisation of sigma and cost parameters through a grid search. For the random forest model (method = ‘rf’ from the ‘randomForest’ package), we optimised the parameter through cross-validation with ‘tuneLength’ = 10.

Model training was conducted using fivefold cross-validation, implemented through the ‘trainControl’ function with method = ‘cv’, number = 5 and ‘classProbs’ = TRUE to enable receiver operating characteristic (ROC) curve calculations. We used the ‘pROC’ package to generate ROC curves and calculate area under the ROC (AUROC). Additional performance metrics were calculated using the ‘confusionMatrix’ function from the ‘caret’ package, with a threshold of 0.5 for class prediction.

We calculated variable importance from the CART model (which achieved the highest ROC value) using the ‘varImp’ function from the ‘caret’ package. This function provides a measure of each variable’s contribution to the model’s predictions, with higher scores indicating greater importance in the prediction process.

We used R (version 3.6.0) for all analyses and defined statistical significance as *P* < 0.05.

### Changes in mortality over time

A retrospective service evaluation was conducted from 2013 to 2019 inclusive, involving four mental health wards for people with dementia in the UK, in order to capture a greater number of events. We examined 1976 admissions, but it is possible that some of these could represent individuals admitted more than once; however, our data suggest that this is a rare event and does not impact the number of people who died while an in-patient. The project was approved as part of a service evaluation by each participating trust. Participating wards provided pre-existing, routinely collected, anonymised data for the calendar years 2013–2019 inclusive. Data provided included the number of deaths as a proportion of admission for each calendar year. Data from the four sites was analysed together, because the number of deaths at any given site per year was low. We analysed the data by fitting a Poisson regression model, including year as a covariate, and compared it with a null model (no change in mortality over time) using a likelihood ratio test to compare the two. Poisson regression was chosen for interpretation of findings as rates. The four participating NHS trusts (Cambridgeshire and Peterborough, Bradford District Care, Leeds and York and Humber Teaching), and the characteristics of their services, have previously been described in detail.^20^

## Results

Of the 576 patients in our cohort, 304 were men (52.8%) and 51.9% were married. The average age at time of admission was 77 years (76.6 ± 8.5). The most common diagnosis was Alzheimer’s disease (64.4%). The median length of in-patient stay was 68 days. Baseline characteristics of the patients are described in Supplementary Table 1.

**Fig. 1:**
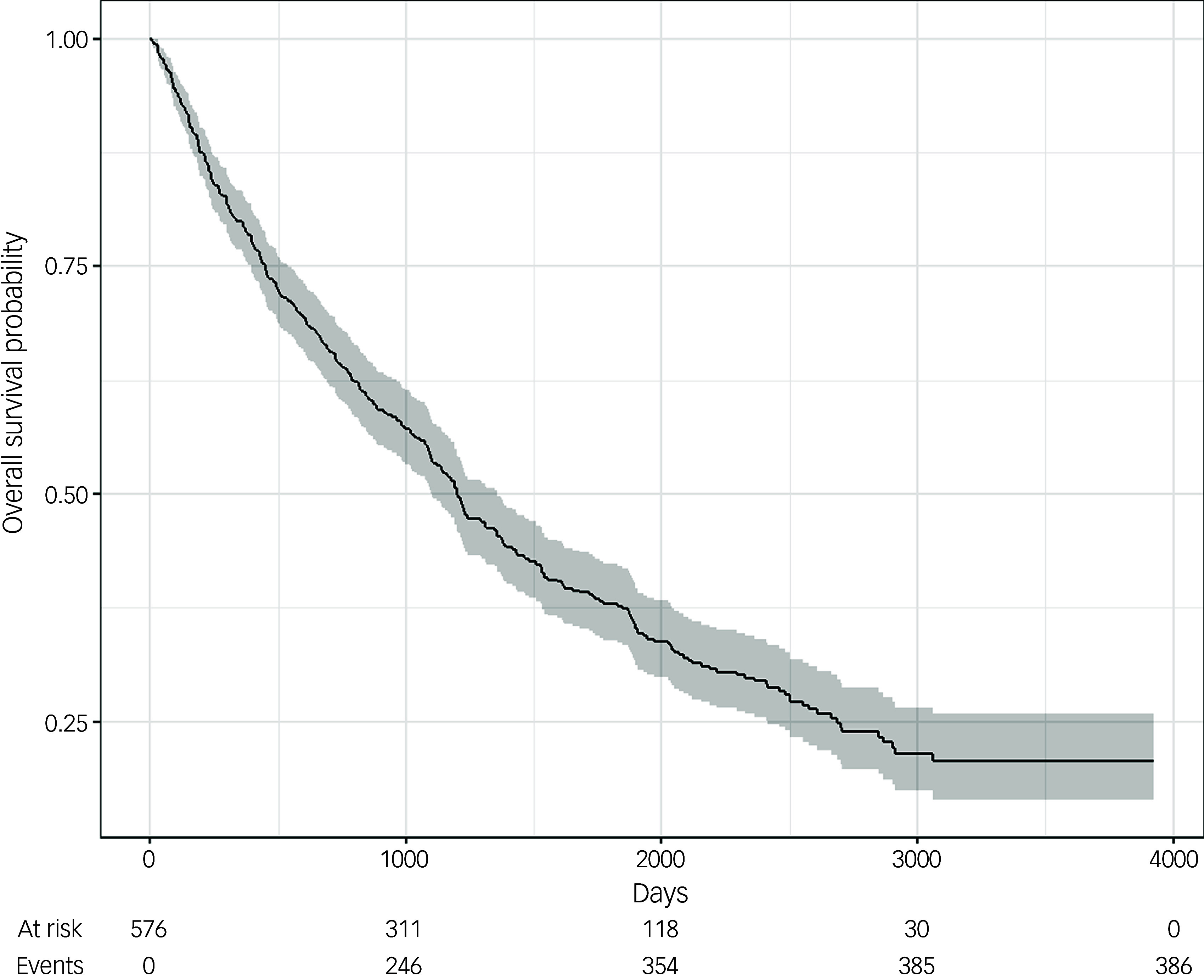
Kaplan–Meier survival curve following admission to a psychiatric ward. Kaplan–Meier survival analysis shows that, following admission to the ward, patients from the cohort had a median survival length of 1201 days (3.3 years). (For reference, 1000 days is 2.7 years, 2000 days is 5.5 years, 3000 days is 8.2 years and 4000 days is 11 years.)

Kaplan–Meier survival analysis showed that median survival length following the date of admission of patients was 1201 days, representing 3.3 years from admission. However, the survival time of patients was variable, with the longest being 3924 days post admission, more than 10 years, showing that not all patients were close to the end of life at the time of admission. Kaplan–Meier survival analysis is shown in Fig. 1.

We next examined potential predictors of mortality. For this, we divided the patient population into two groups: those who died within 1 year of admission and those who lived beyond 1 year. There were 119 patients out of 576 (20.7%) who died within 1 year of admission. We identified several variables significantly associated with these mortality outcomes, including age, gender, HoNOS disability category and specific medications. Supplementary Table 2 summarises the statistical comparison between groups.

With regard to age, a two-sample Mann–Whitney *U*-test (*P* < 0.001) showed a significant difference between the two groups. People who died within 1 year of admission were on average older at the time of admission (80 [10.5] years) compared with those who lived longer (77 [12] years). Similarly, sex assigned at birth was associated with patient outcome (*P* < 0.001), and a higher percentage of men was found in the group of patients who died within 1 year of admission. We examined HoNOS scores and, despite finding no significant difference between the total scores of both groups, we noted a difference in an individual category: an outcome of death within 1 year was associated with a higher median disability score (2) compared with patients who survived longer (1) (*P* = 0.009). A prescription of citalopram was associated with the examined outcome (*P* = 0.04); the group that survived longer was associated with a higher percentage of prescriptions. There was no significant difference (*P* = 0.06) in the total number of lifetime prescriptions between patients who died within 1 year of admission (19 [13] years) compared with those who lived longer (21 [15] years). Similarly, we did not identify any significant differences between groups regarding marital status, IMD, diagnosis or cognitive tests.

**Fig. 2:**
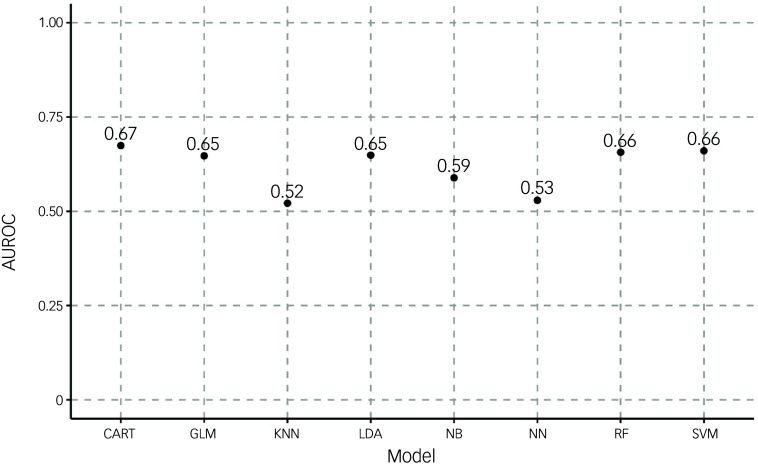
Area under the receiving operator curve (AUROC) for models predicting death within 1 year. AUROC was calculated for eight different algorithms (classification and regression trees (CART), generalised linear model (GLM), *k*-nearest neighbours (KNN), linear discriminant analysis (LDA), naive Bayesian (NB), neural network (NN), random forest (RF) and support vector machine (SVM)) for outcomes of either death within 1 year or no death.

Because we were able to identify significant differences between the high- and low-mortality risk groups, we moved on to produce eight machine learning models aimed at predicting death within 1 year of arrival on the ward, which is a criterion for palliative care. Supplementary Table 3 shows the balance of the train and test datasets used for machine learning. Figure 2 indicates that machine learning models showed varying levels of performance in predicting mortality within 1 year of admission. In terms of discrimination ability as measured by AUROC, the CART model achieved the highest performance (0.67), followed by random forest and SVM (both 0.66) and logistic regression and LDA (both 0.65). The KNN model showed the weakest performance, with an AUROC of 0.52, while neural network and naive Bayes models showed moderate performance, with AUROC of 0.53 and 0.59, respectively. Both CART and logistic regression models achieved the highest precision (0.82), indicating that they had the lowest false-positive rates among all models (Supplementary Fig. 2). For recall (sensitivity) (Supplementary Fig. 3), the random forest model notably outperformed other approaches, with a recall of 0.99, although this high recall came at the cost of reduced precision (0.76). Other models showed more balanced recall values, ranging from 0.72 (naive Bayes) to 0.81 (LDA).

The variable importance analysis from the CART model revealed that male gender and total number of drug prescriptions were the two most influential predictors, followed by age at admission and disability score from HoNOS (Supplementary Fig. 4). Other important predictors included readmission to a DMHW and subscales from HoNOS (activities of daily living (ADL) and behaviour). Notably, specific medication prescriptions, cognitive test scores and most other HoNOS subscales showed relatively lower importance in predicting mortality.

**Fig. 3:**
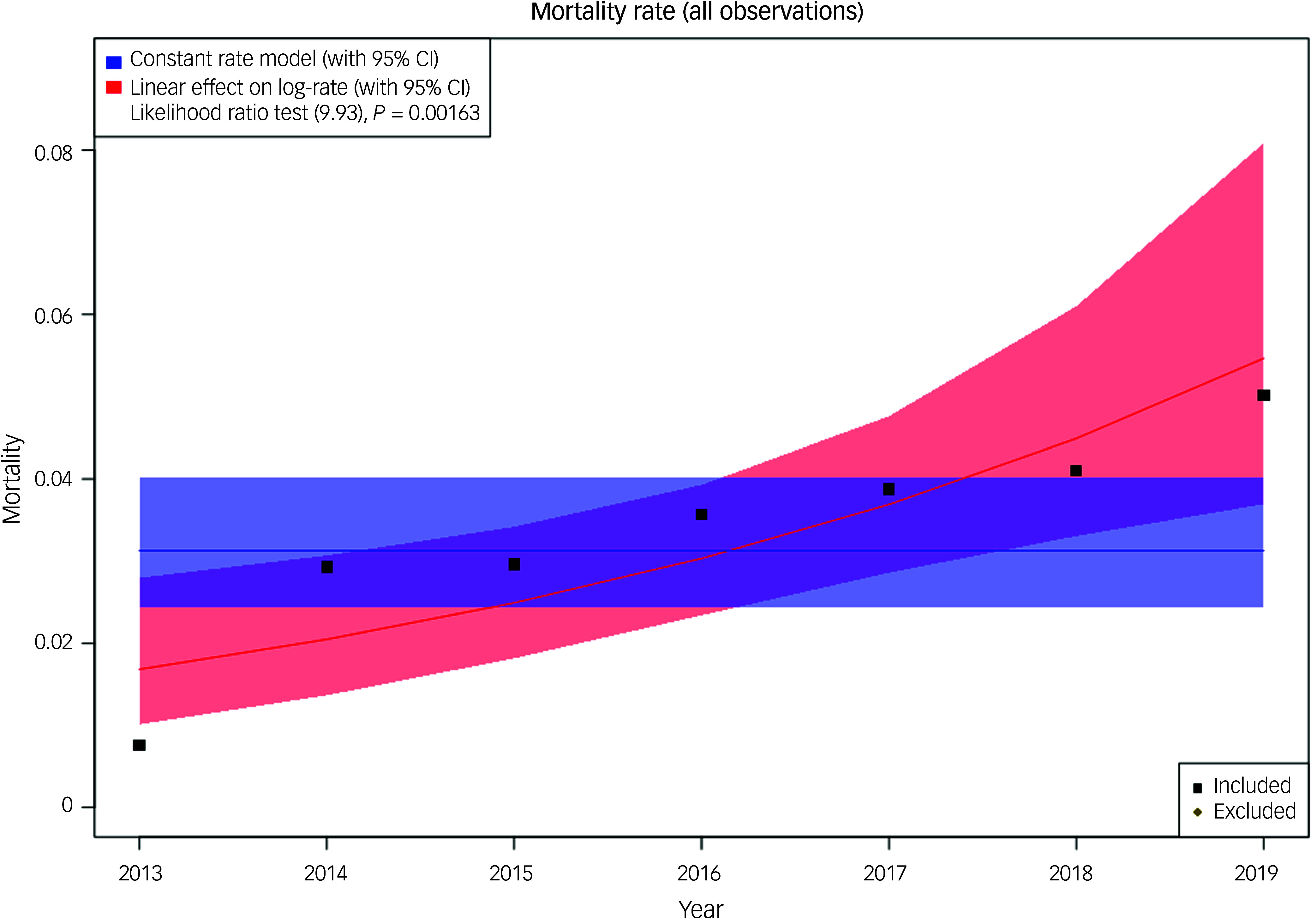
Mortality rate per admission against time in four wards. The plot includes the observed data (as deaths/admissions), the null model (blue) and the year-related model (red). Model fits are shown with 95% CIs. The likelihood ratio test (chi-squared 9.93 on 1 degree of freedom, *P* = 0.0016) supports year as a covariate in the model. All modelling techniques used found a statistically significant association between year and mortality, with the latter as a proportion of admissions increasing over time. Because Poisson regression models the log-rate, we have included calendar year as a linear covariate in the log-rate model; hence, when plotting the rate (the exponential of log-rate) we see a non-linear trend.

Finally, using data obtained from four different wards around the UK, a retrospective study service evaluation was conducted to investigate changes in on-ward deaths over time. The four sites serve varying population sizes and had differing numbers of beds available (12–21). In relation to the care of people who were dying, there was clear variance in the staffing and resources available: only two of the wards had access to consultants in geriatric medicine, and one ward employed registered general nurses alongside registered mental health nurses. All wards were able to refer to palliative care teams if required. Over the 7-year period analysed, there were 1976 admissions and 61 deaths recorded as occurring on these wards. We investigated whether the number of deaths on the wards had changed over time, and the mortality rate per admission against time is shown in Fig. 3. Our data show evidence of an increase in on-ward mortality over time, rising from 3 deaths per year in 2013 to 13 in 2019.

## Discussion

There has been little research on mortality for PLWD in DMHWs. To the best of our knowledge, this is the first study to report a survival time for PLWD admitted in DMHWs. In our cohort, we found significant but variable mortality following admission: 20.7% of PLWD died within 1 year of admission, and the median survival length of 1201 days indicated that around half were in their final 3 years of life at the point of admission. However, some patients survived for a much longer time. Our data are consistent with a multicentre cohort study from France that reported 16.7% of participants dying within 1 year following discharge from cognitive behavioural units, specialised wards for patients with BPSD.^21^ Given the terminal nature of the condition and the association of BPSD symptoms with advanced disease, the number of people surviving for prolonged periods of time (half for 3 years, some for as long as 10) is perhaps surprising. Our data underline the variability in prognosis for dementia and the potential for prolonged survival with good nursing care, even in advanced disease.

We were able to identify certain characteristics associated with an increased risk of death, including male sex and age. The machine learning models, built using data available from electronic patient records at the point of admission and incorporating these factors, were not able to predict patients likely to die within 1 year of admission to a degree of accuracy that would probably render them clinically useful – for example, by stratifying those being admitted in terms of risk of mortality and subsequently allowing targeting of any interventions. It is possible that our difficulty in identifying those at highest risk of mortality reflects the importance of other factors in influencing this – for example, events such as fall to fracture or infection. Interestingly, one study conducted on mortality for PLWD in acute hospital care settings developed a logistic regression model to predict patients with advanced dementia at risk of dying within 1 year.^14^ In that study, advanced age and male gender were found to be predictors of 1-year mortality, which fits with what was observed in our cohort; however, it found additional predictors related to health such as pressure ulcers, dysphagia, pneumonia and more. Thus, our machine learning models might benefit from further clinical variables. Additionally, the variation in machine learning model performance may be due to the smaller sample size of our cohort, or to the fact that the patient group differed between the two studies. In particular, patients in general hospitals had a higher mortality rate. Given the uncertainty about the best point in the care pathway at which to have conversations around advanced care planning, the variable mortality following admission and the difficulty in identifying any particularly high-risk group, we suggest admission as an important inflection point in care that provides an opportunity to explore this issue with all PLWD admitted, and their families.

We were specifically interested in the number of deaths occurring on specialist dementia wards and how this was changing over time, because it has relevance for the availability of palliative care approach in these settings, something we have previously identified as being variable.^20^ We found that such deaths were still relatively rare, and therefore needed to combine data from more than one site to examine trends over time. We found that the number of deaths occurring during admission to DMHWs has increased over time. The number of psychiatric beds available in the UK has significantly decreased in the last 20 years, from more than 54 000 in 2000 to around 23 000 in 2021. This national picture has been mirrored in Cambridgeshire, where there are currently four wards for older people compared with eight wards and two day-hospitals 20 years ago. This may have impacted mortality, because a reduction in bed numbers would require prioritisation of patients presenting with more severe symptoms. Whatever the cause, the increase in on-ward deaths underlines the need for appropriate palliative care to be available on DMHWs.

Palliative support can be provided in several ways, such as linking the wards more closely with palliative care teams or upskilling existing staff on the ward. However, palliative care for people with dementia is a relatively poorly understood clinical area compared with other common causes of death, such as cancer. Given the complexity of patients on these wards due to their specific psychological needs, a specialist service may be appropriate – or, at the very least, collaboration between clinicians with the different skills necessary to manage this complex clinical challenge.^22^ The data presented here should help inform end-of-life conversations and make the case for their importance in this group of people. Developing better palliative care settings for PLWD is especially important given the predicted rise in need for such services, associated with the increased prevalence of dementia that is estimated to reach 1.2 million people in the UK by 2025.^23^

Our study has a number of limitations. First, in our study design, while we recorded admissions only until 2019, the follow-up lasted until 2023, which means that there might have been an impact of the COVID-19 pandemic on mortality that could have affected our machine learning predictions. The analysis of death following admission, and the attempts to identify clinical features associated with mortality, are based on a retrospective cohort design that can only describe associations without inferring causation, and are naturalistic data not collected for the specific purpose of analysis. These data were collected at a single site only and our machine learning results are not externally validated, although the lack of significantly predictive data would mean that any differences seen at other sites would infer significant heterogeneity, which would limit the applicability of any predictive models that might be identified. The increase in on-ward mortality was seen in the combined data from four different NHS trusts. While these cover a variety of rural and urban settings and heterogeneous populations, we cannot rule out specific local issues driving the overall trend. Additionally, our analysis covered a 7-year period only and we cannot exclude the possibility that mortality had been fluctuating over longer periods. Last, we did not have data on the causes of death in this group.

Our study highlights a variable and unpredictable mortality rate for PLWD admitted to mental health wards, and an increase in deaths occurring on these wards over time. Admission may be a point in the care pathway where end-of-life provision can be discussed or revisited, and appropriate palliative care provision should be available in in-patient settings. How this is best done and provided is something that should be developed with PLWD and their families.

## Supporting information

Marguet et al. supplementary material Marguet et al. supplementary material

## Data Availability

The anonymised data underpinning this project are available on reasonable request, subject to any restrictions arising from UK data protection law.

## Supplementary material

The supplementary material is available online at https://doi.org/10.1192/bjo.2025.40
