# Supplementary material for: Mortality and its predictors among people with dementia receiving psychiatric in-patient care
Source: BJPsych Open. 2025 May 9;11(3):e92. doi: 10.1192/bjo.2025.40 (PMC12089802; doi:10.1192/bjo.2025.40)
Supplement: Marguet et al. supplementary material [file S2056472425000407sup001.docx]

**Supplementary materials**

**Supplementary Table 1. Cohort characteristics**

| Variable | Cohort characteristics (n=576) |
| --- | --- |
| Gender  Male  Female | 52.8%  47.2% |
| Age (Mean ± SD) | 76.6 ± 8.5 |
| Marital Status  Married  Cohabiting  Civil Partnership  Divorced  Separated  Single  Widowed  *NA* | 51.91%  0.52%  0.52%  5.38%  1.56%  4.69%  24.83%  10.69% |
| Ethnicity  White  Mixed  Asian  Black  Other  Unknown | 89.58%  0.69%  1.91%  0.17%  0.69%  6.94% |
| Total HoNOS score (Mean ± SD)  Individual HoNOS categories (Mode)  Behaviour  Selfharm  Subs (Substances)  Cognitive  Disability  Hallucinations  Depressed  Other  Relationships  ADL  Living Conditions  Occupation | 13.5 ± 5.5  1  1  0  2  0  0  0  0  0  2  0  0 |
| Cognitive Test Scores (Mean ± SD)  ACE  Mini-ACE  MMSE | 61.3 ± 17.9  13.4 ± 8.1  19.7 ± 6.5 |
| IMD Deciles (Mode) | 6 |
| ICD-10 Diagnosis code  Alzheimer’s disease (F00-, G30-)  Vascular dementia (F01)  Other dementia (F02)  Unspecified dementia (F03) | 64.41%  18.23%  7.99%  9.38% |
| Prescriptions*  Donepezil  Rivastigmine  Galantamine  Memantine  Risperidone  Mirtazapine  Citalopram  Sertraline  Trazodone | 39.41%  16.67%  6.08%  57.81%  59.55%  46.70%  30.03%  23.78%  6.77% |

*Table 1. Baseline characteristics of the cohort.*

**”Prescriptions” refer to whether the patient had been prescribed a drug from the following list in their lifetime before admission to the DMHW.*

**Supplementary Table 2. Comparison between the two groups.**

| Variable | No death within a year (n=457) | Death within a year (n=119) | P-value |
| --- | --- | --- | --- |
| Gender  Male  Female | 48%  52% | 42.2%  57.8% | < 0.001 *** |
| Age (Mean ± SD) | 75.9 **±** 8.67 | 79.0 **±** 7.51 | < 0.001 *** |
| Marital Status  Married  Cohabiting  Civil Partnership  Divorced  Separated  Single  Widowed  *NA* | 50.3%  0.7%  0.4%  6.1%  1.5%  5.1%  25.8%  10.1% | 58%  0%  0.8%  2.5%  1.7%  3.4%  21%  12.6% | 0.46 |
| Total HoNOS score (Mean ± SD)  Individual HoNOS categories (Mode)  Behaviour  Selfharm  Subs (Substances)  Cognitive  Disability  Hallucinations  Depressed  Other  Relationships  ADL  Living Conditions  Occupation | 13.4 ± 5.48  1  1  0  2  0  0  0  0  0  1  0  0 | 13.9 + 5.82  1  1  0  2  2  0  0  0  0  2  0  0 | 0.55  0.44  0.13  0.62  0.22  0.009 **  0.42  0.78  0.83  0.53  0.84  0.90  0.95 |
| Cognitive Test Scores (Mean ± SD)  ACE  Mini-ACE  MMSE | 60.5 ± 18.4  10.8 ± 7.97  19.8 ± 6.55 | 66.0 ± 14.0  10.0 ± 10.0  19.2 ± 6.70 | 0.20  0.61  0.61 |
| IMD Deciles (Mode) | 6 | 6, 7 | 0.89 |
| ICD-10 Diagnosis code  Alzheimer’s disease (F00-, G30-)  Vascular dementia (F01)  Other dementia (F02)  Unspecified dementia (F03) | 63%  18.9%  8.5%  9.6% | 69.7%  16%  5.9%  8.4% | 0.56 |
| Lifelong prescription  Donepezil  Rivastigmine  Galantamine  Memantine  Risperidone  Mirtazapine  Citalopram  Sertraline  Trazodone | 39.8%  16.2%  5.7%  58.2%  62.3%  48.4%  32.2%  25.6%  7.2% | 37.8%  18.5%  7.6%  56.3%  52.9%  40.3%  21.8%  16.8%  5% | 0.77  0.65  0.58  0.79  0.12  0.14  0.03 *  0.06 *  0.53 |

*The table summarises statistical differences between the two groups. P-values were obtained after t-tests or Mann-Whitney U tests when appropriate for continuous variables, chi-squared tests or Fisher’s exact tests when appropriate for categorical variables, and Mann-Whitney U tests were used for ordinal variables.*

**Supplementary Table 3. Comparison between testing and training datasets for ML model.**

| Variable | dataset (= Test) | dataset (= Train) | dataset (= All) | test_name | test_value | p_value |
| --- | --- | --- | --- | --- | --- | --- |
| Age at admission | 79.08 (8.14) | 79.71 (7.94) | 79.62 (7.97) | t.test | -0.76054 | 0.448102 |
| Gender (= Female) | 61 (53.5%) | 325 (49.7%) | 386 (50.3%) | chisq.test | 0.422764 | 0.515561 |
| Gender (= Male) | 53 (46.5%) | 329 (50.3%) | 382 (49.7%) | chisq.test | 0.422764 | 0.515561 |
| Marital status (= TRUE) | 54 (47.4%) | 363 (55.5%) | 417 (54.3%) | chisq.test | 3.37571 | 0.184916 |
| Marital status (= FALSE) | 48 (42.1%) | 218 (33.3%) | 266 (34.6%) | chisq.test | 3.37571 | 0.184916 |
| Marital status (= Not known) | 12 (10.5%) | 73 (11.2%) | 85 (11.1%) | chisq.test | 3.37571 | 0.184916 |
| Ethnicity (= White) | 107 (93.9%) | 575 (87.9%) | 682 (88.8%) | chisq.test | 3.646801 | 0.161476 |
| Ethnicity (= Others) | 1 (0.9%) | 19 (2.9%) | 20 (2.6%) | chisq.test | 3.646801 | 0.161476 |
| Ethnicity (= Unknown) | 6 (5.3%) | 60 (9.2%) | 66 (8.6%) | chisq.test | 3.646801 | 0.161476 |
| IMD (= Most deprived) | 11 (9.6%) | 80 (12.2%) | 91 (11.8%) | chisq.test | 12.06091 | 0.209897 |
| IMD (= 2) | 14 (12.3%) | 95 (14.5%) | 109 (14.2%) | chisq.test | 12.06091 | 0.209897 |
| IMD (= 3) | 17 (14.9%) | 71 (10.9%) | 88 (11.5%) | chisq.test | 12.06091 | 0.209897 |
| IMD (= 4) | 12 (10.5%) | 88 (13.5%) | 100 (13.0%) | chisq.test | 12.06091 | 0.209897 |
| IMD (= 5) | 19 (16.7%) | 105 (16.1%) | 124 (16.1%) | chisq.test | 12.06091 | 0.209897 |
| IMD (= 6) | 12 (10.5%) | 66 (10.1%) | 78 (10.2%) | chisq.test | 12.06091 | 0.209897 |
| IMD (= 7) | 11 (9.6%) | 30 (4.6%) | 41 (5.3%) | chisq.test | 12.06091 | 0.209897 |
| IMD (= 8) | 10 (8.8%) | 55 (8.4%) | 65 (8.5%) | chisq.test | 12.06091 | 0.209897 |
| IMD (= 9) | 4 (3.5%) | 53 (8.1%) | 57 (7.4%) | chisq.test | 12.06091 | 0.209897 |
| IMD (= Least deprived) | 4 (3.5%) | 11 (1.7%) | 15 (2.0%) | chisq.test | 12.06091 | 0.209897 |
| Dementia diagnosis (= Alzheimer's disease) | 72 (63.2%) | 437 (66.8%) | 509 (66.3%) | chisq.test | 2.398058 | 0.493996 |
| Dementia diagnosis (= Other dementia) | 9 (7.9%) | 49 (7.5%) | 58 (7.6%) | chisq.test | 2.398058 | 0.493996 |
| Dementia diagnosis (= Unspecified dementia) | 14 (12.3%) | 52 (8.0%) | 66 (8.6%) | chisq.test | 2.398058 | 0.493996 |
| Dementia diagnosis (= Vascular dementia) | 19 (16.7%) | 116 (17.7%) | 135 (17.6%) | chisq.test | 2.398058 | 0.493996 |
| MMSE | 62.85 (21.51) | 60.72 (22.58) | 61.04 (22.42) | t.test | 0.969174 | 0.333925 |
| ACE | 56.52 (21.30) | 56.82 (20.76) | 56.77 (20.83) | t.test | -0.14115 | 0.887934 |
| Mini-ACE | 38.89 (28.45) | 37.20 (29.05) | 37.45 (28.95) | t.test | 0.582382 | 0.561146 |
| HoNOS Behaviour | 2.12 (1.16) | 2.25 (1.19) | 2.23 (1.19) | t.test | -1.056 | 0.292587 |
| HoNOS SelfHarm | 1.15 (0.52) | 1.09 (0.38) | 1.10 (0.41) | t.test | 1.127289 | 0.261614 |
| HoNOS Subs | 0.07 (0.29) | 0.09 (0.39) | 0.08 (0.37) | t.test | -0.49861 | 0.618625 |
| HoNOS Cognitive | 2.11 (0.97) | 2.20 (0.96) | 2.19 (0.96) | t.test | -0.86442 | 0.388695 |
| HoNOS Disability | 1.35 (1.17) | 1.37 (1.09) | 1.37 (1.10) | t.test | -0.20244 | 0.839851 |
| HoNOS Hallucinations | 0.89 (1.21) | 0.76 (1.04) | 0.78 (1.07) | t.test | 1.154455 | 0.250228 |
| HoNOS Depressed | 0.73 (0.91) | 0.76 (0.89) | 0.76 (0.89) | t.test | -0.39418 | 0.693999 |
| HoNOS Other | 1.32 (1.20) | 1.22 (1.20) | 1.23 (1.20) | t.test | 0.822567 | 0.412016 |
| HoNOS Relationships | 0.97 (0.99) | 0.92 (1.05) | 0.93 (1.04) | t.test | 0.494424 | 0.621684 |
| HoNOS ADL | 1.75 (1.05) | 1.85 (1.13) | 1.83 (1.12) | t.test | -0.85811 | 0.392098 |
| HoNOS Living Conditions | 0.60 (0.95) | 0.44 (0.83) | 0.46 (0.85) | t.test | 1.636701 | 0.103867 |
| HoNOS Occupation | 0.86 (0.94) | 0.82 (0.95) | 0.82 (0.95) | t.test | 0.435809 | 0.663579 |
| Readmission (= 0) | 89 (78.1%) | 557 (85.2%) | 646 (84.1%) | chisq.test | 3.14844 | 0.076 |
| Readmission (= 1) | 25 (21.9%) | 97 (14.8%) | 122 (15.9%) | chisq.test | 3.14844 | 0.076 |
| Donepezil (= FALSE) | 73 (64.0%) | 394 (60.2%) | 467 (60.8%) | chisq.test | 0.437005 | 0.508571 |
| Donepezil (= TRUE) | 41 (36.0%) | 260 (39.8%) | 301 (39.2%) | chisq.test | 0.437005 | 0.508571 |
| Rivastigmine (= FALSE) | 97 (85.1%) | 545 (83.3%) | 642 (83.6%) | chisq.test | 0.108722 | 0.741604 |
| Rivastigmine (= TRUE) | 17 (14.9%) | 109 (16.7%) | 126 (16.4%) | chisq.test | 0.108722 | 0.741604 |
| Galantamine (= FALSE) | 106 (93.0%) | 613 (93.7%) | 719 (93.6%) | chisq.test | 0.008852 | 0.925041 |
| Galantamine (= TRUE) | 8 (7.0%) | 41 (6.3%) | 49 (6.4%) | chisq.test | 0.008852 | 0.925041 |
| Memantine (= FALSE) | 49 (43.0%) | 280 (42.8%) | 329 (42.8%) | chisq.test | 0 | 1 |
| Memantine (= TRUE) | 65 (57.0%) | 374 (57.2%) | 439 (57.2%) | chisq.test | 0 | 1 |
| risperidone (= FALSE) | 40 (35.1%) | 285 (43.6%) | 325 (42.3%) | chisq.test | 2.529539 | 0.111733 |
| risperidone (= TRUE) | 74 (64.9%) | 369 (56.4%) | 443 (57.7%) | chisq.test | 2.529539 | 0.111733 |
| mirtazapine (= FALSE) | 57 (50.0%) | 370 (56.6%) | 427 (55.6%) | chisq.test | 1.444072 | 0.229482 |
| mirtazapine (= TRUE) | 57 (50.0%) | 284 (43.4%) | 341 (44.4%) | chisq.test | 1.444072 | 0.229482 |
| citalopram (= FALSE) | 81 (71.1%) | 468 (71.6%) | 549 (71.5%) | chisq.test | 0 | 1 |
| citalopram (= TRUE) | 33 (28.9%) | 186 (28.4%) | 219 (28.5%) | chisq.test | 0 | 1 |
| sertraline (= FALSE) | 91 (79.8%) | 506 (77.4%) | 597 (77.7%) | chisq.test | 0.210982 | 0.645999 |
| sertraline (= TRUE) | 23 (20.2%) | 148 (22.6%) | 171 (22.3%) | chisq.test | 0.210982 | 0.645999 |
| trazodone (= FALSE) | 105 (92.1%) | 614 (93.9%) | 719 (93.6%) | chisq.test | 0.259451 | 0.610498 |
| trazodone (= TRUE) | 9 (7.9%) | 40 (6.1%) | 49 (6.4%) | chisq.test | 0.259451 | 0.610498 |
| Total number of drug prescriptions | 23.52 (14.10) | 22.68 (10.52) | 22.80 (11.12) | t.test | 0.6065 | 0.545195 |

**Supplementary Figure 1. Flow chart of patients included in the study.**


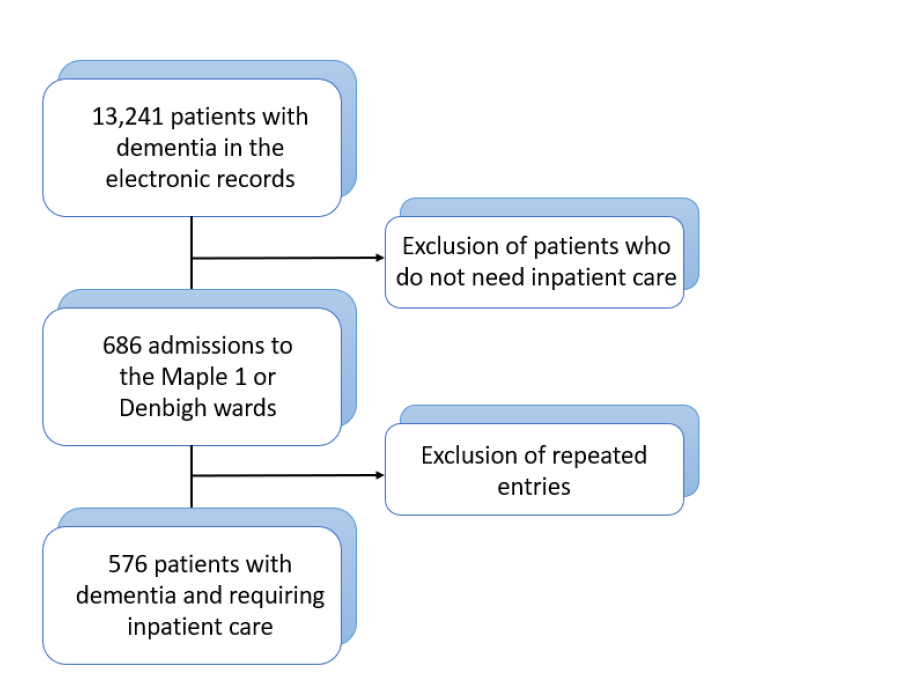


**Supplementary Figure 2. Precision of machine learning models.**

**
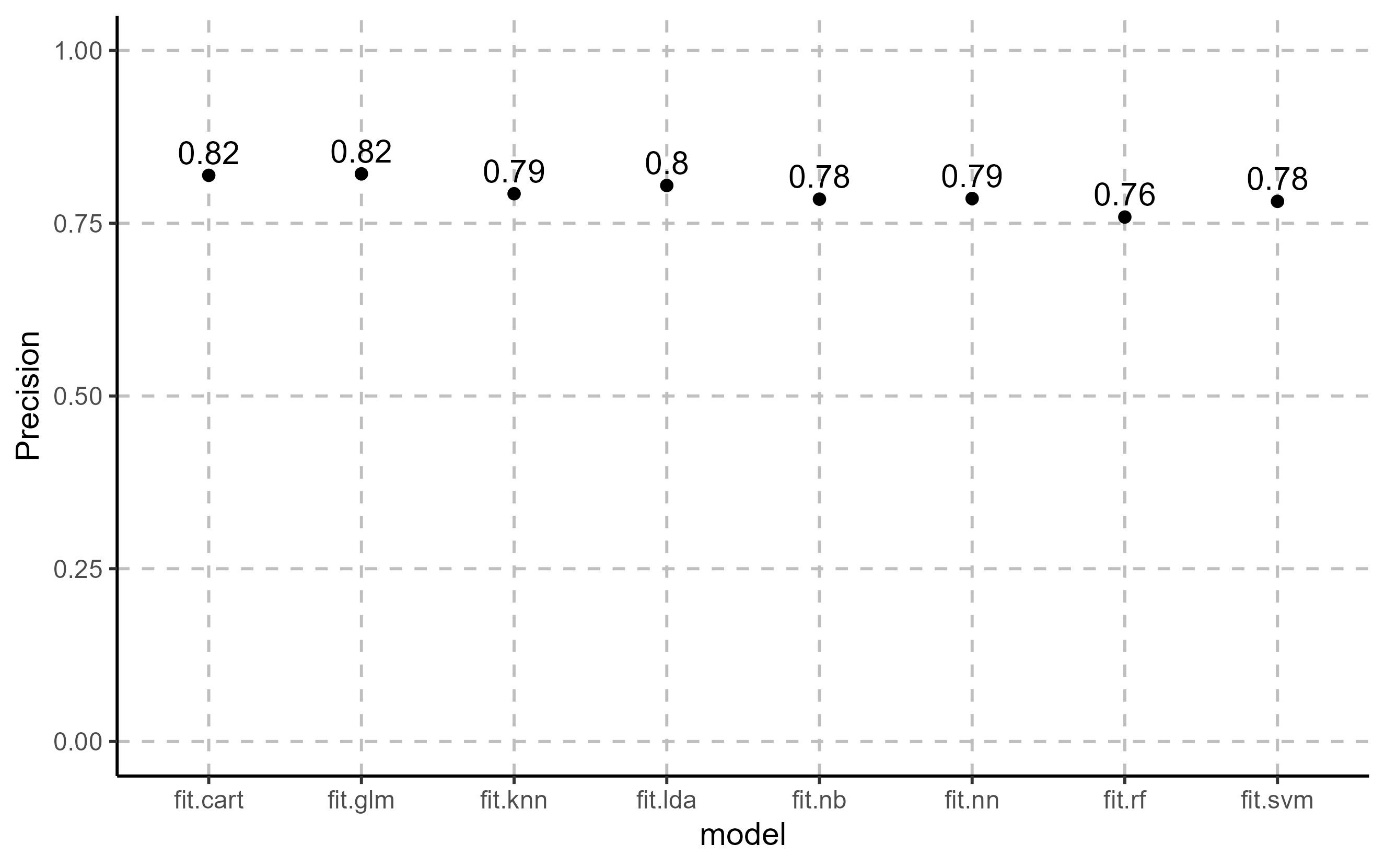
**

**Supplementary Figure 3. Recall of machine learning models**

**
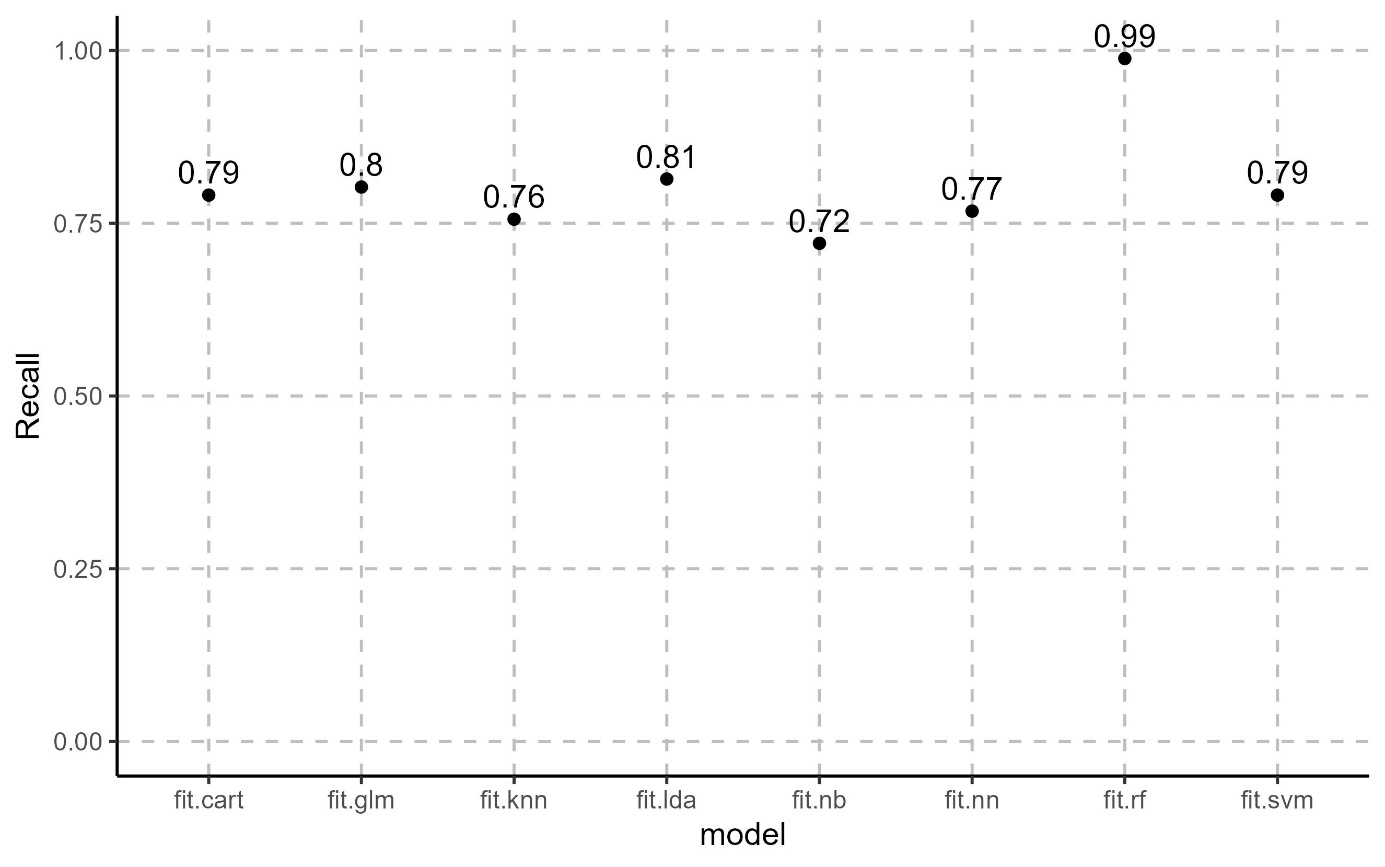
**

**Supplementary Figure 4. Variable importance on the prediction.**

**
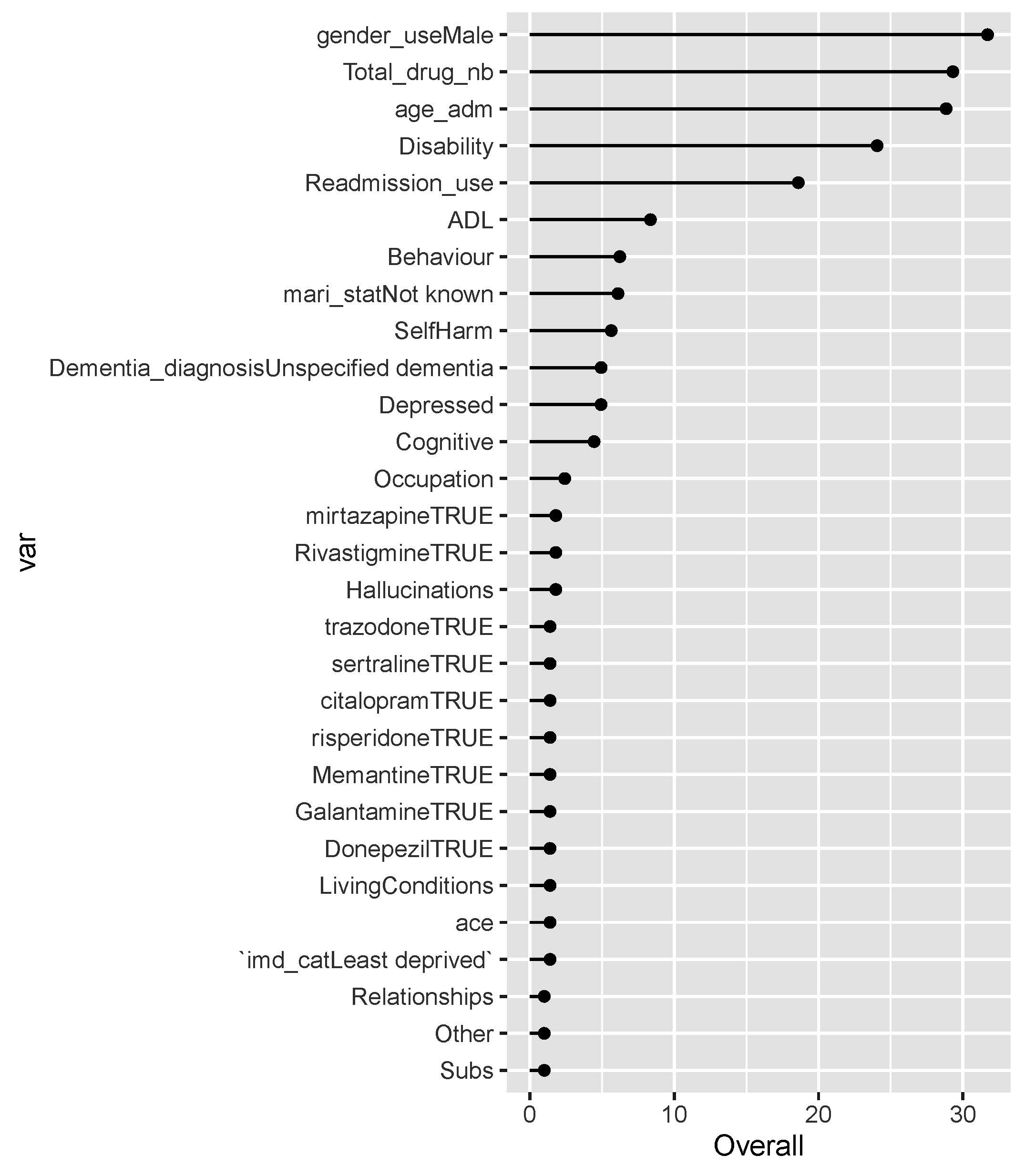
**

*Supplementary Figure 4. Ranking of variables used in the ML model in order of importance. Signification of variable names: “gender_useMale” = gender (category male); ”Total_drug_nb” = total number of drug prescriptions; “age_adm” = age at admission; “Disability”, “ADL”, “Behaviour”, “SelfHarm”, “Depressed”, “Cognitive”, “Occupation”, “Hallucinations” “LivingConditions”, “Other”, “Subs” = HoNOS subcategories; “Readmission_use” = readmission status of patient; “mari_statUnknown” = marital status (category unknown); “Dementia_diagnosisUnspecified dementia” = diagnosis (category unspecified dementia); “trazodoneTRUE”, “sertralineTRUE”, “citalopramTRUE”, risperidoneTRUE, “MemantimeTRUE”, “GalantamineTRUE”, “DonepeziTRUE” = prescription of the corresponding drug; “ace” = score on the ACE; “imd_catLeastdeprived” = IMD (category least deprived).*
